# A comparative *in vitro* study on the effect of SGLT2 inhibitors on chemosensitivity to doxorubicin in MCF-7 breast cancer cells

**DOI:** 10.32604/or.2024.048988

**Published:** 2024-04-23

**Authors:** SHAHID KARIM, ALANOUD NAHER ALGHANMI, MAHA JAMAL, HUDA ALKREATHY, ALAM JAMAL, HIND A. ALKHATABI, MOHAMMED BAZUHAIR, AFTAB AHMAD

**Affiliations:** 1 Department of Clinical Pharmacology, Faculty of Medicine, King Abdulaziz University, Jeddah, Saudi Arabia; 2 Department of Biochemistry, Faculty of Science, King Abdulaziz University, Jeddah, Saudi Arabia; 3 Department of Biochemistry, College of Science, University of Jeddah, Jeddah, 21959, Saudi Arabia; 4 Health Information Technology Department, The Applied College, King Abdulaziz University, Jeddah, Saudi Arabia; 5 Pharmacovigilance and Medication Safety Unit, Center of Research Excellence for Drug Research and Pharmaceutical Industries, King Abdulaziz University, Jeddah, Saudi Arabia

**Keywords:** SGLT2, Cancer, Cytotoxicity, ATP, Cell cycle

## Abstract

Cancer frequently develops resistance to the majority of chemotherapy treatments. This study aimed to examine the synergistic cytotoxic and antitumor effects of SGLT2 inhibitors, specifically Canagliflozin (CAN), Dapagliflozin (DAP), Empagliflozin (EMP), and Doxorubicin (DOX), using *in vitro* experimentation. The precise combination of CAN+DOX has been found to greatly enhance the cytotoxic effects of doxorubicin (DOX) in MCF-7 cells. Interestingly, it was shown that cancer cells exhibit an increased demand for glucose and ATP in order to support their growth. Notably, when these medications were combined with DOX, there was a considerable inhibition of glucose consumption, as well as reductions in intracellular ATP and lactate levels. Moreover, this effect was found to be dependent on the dosages of the drugs. In addition to effectively inhibiting the cell cycle, the combination of CAN+DOX induces substantial modifications in both cell cycle and apoptotic gene expression. This work represents the initial report on the beneficial impact of SGLT2 inhibitor medications, namely CAN, DAP, and EMP, on the responsiveness to the anticancer properties of DOX. The underlying molecular mechanisms potentially involve the suppression of the function of SGLT2.

## Introduction

Cancer is responsible for the mortality of approximately 16% of the global population, positioning it as the second most prevalent cause of death following heart disease [1]. Currently, the primary methods for treating malignancies or malignant tumors involve surgical resection, chemotherapy, radiotherapy and immunotherapy. The immunotherapy technique of chimeric antigen receptor (CAR)-T cells has resulted in significant clinical outcomes when treating hematologic malignancies. The immunosuppressive microenvironment of solid tumors, the shortage of tumor-specific antigens, and post-treatment side effects are the major hindrances to promoting the development of CAR-T cells. Several clinical trials related to CAR-T immunotherapy against CRC or BC have already been in progress [2]. Mitochondrial DNA (mtDNA) is an emerging and fast-developing field of research. It has been demonstrated that cancer cells contain ROS/oxidative stress-mediated defects in the mtDNA repair system and mitochondrial nucleoid protection. Accordingly, several mitochondria-targeting therapeutic agents (biguanides, OXPHOS inhibitors, vitamin-E analogues, and antibiotic bedaquiline) were suggested for future clinical trials in breast cancer patients [3]. However, crosstalk mechanisms between altered mitoepigenetics and cancer-associated mtDNA mutations remain largely unclear [4]. Among the several therapeutic modalities available, chemotherapy has emerged as a very efficacious approach for managing the uncontrolled growth of malignant cells within an organism. Nevertheless, chemotherapy frequently exhibits pronounced adverse effects.

Numerous mechanisms of resistance to DOX have been identified in the literature. These include multidrug resistance facilitated by ATP-binding cassette transporters [5], loss of topoisomerase function, perturbations in DNA damage response and repair pathways, acquisition of cancer stemness properties, overexpression of P-gp [6], and activation of autophagy [7]. These factors play a crucial role in mediating resistance to DOX. Nevertheless, the development of an optimal pharmacological agent capable of effectively addressing chemotherapy resistance remains an ongoing challenge.

In order to sustain their rapid proliferation, cancer cells exhibit a heightened reliance on glucose for glycolysis and energy generation. One of the transporters responsible for facilitating active glucose uptake into cancer cells is known as sodium–glucose cotransporter 2 (SGLT2), which has been identified in different types of tumors [8]. The potential therapeutic application of SGLT2 inhibitors (SGLT2i), a class of antidiabetic drugs, in the treatment of certain cancers has been investigated based on the observed protein expression and functional activity of SGLT2 in different tumor types [9]. The underlying hypothesis is that inhibiting glucose transport into cancer cells through SGLT2 inhibition may have the potential to restrict tumor growth and proliferation [10].

Canagliflozin (CAN), Dapagliflozin (DAP), and Empagliflozin (EMP) are classified as inhibitors of sodium–glucose cotransporter 2 (SGLT2) and have been found to possess a noteworthy hypoglycemic impact [11]. Recent research has indicated that these drugs demonstrate anticancer properties [12]. The study conducted by Shoda et al. provided confirmation that glioma cells rely on SGLT2 for the uptake of glucose [13]. Furthermore, the study demonstrated that the SGLT2 inhibitor canagliflozin effectively inhibits the glucose uptake of these cells. Another study revealed that canagliflozin effectively hinders the growth of Huh7 and HepG2 cells via the suppression of glucose uptake, lactate formation, and intracellular ATP synthesis [14]. Empagliflozin (EMP), which is a sodium–glucose cotransporter 2 (SGLT2) inhibitor, has been documented to exhibit inhibitory properties towards various types of cancer cells, including lung cancer, breast cancer, cervical cancer, and hepatocellular carcinoma (HCC) [12]. One potential molecular mechanism involves the inhibition of glucose reabsorption by the renal tubules. This leads to a reduction in the availability of glucose, which is essential for the growth and metabolism of tumor cells. Consequently, the growth and proliferation of tumor cells are hindered [15]. Nevertheless, initial experiments have suggested that the direct anticancer efficacy of these medications is limited. We believe that these medications may either reduce tumor cells’ resistance to antitumor medications or make antitumor medications more lethal to tumor cells.

This work aimed to examine the impact of the co-administration of CAN and DOX, DAP and DOX, and EMP and DOX on tumor cells in an *in vitro* setting. Additionally, we assessed the cytotoxicity of these combinations in MCF-7 cells. Furthermore, we assessed the impact of these factors on the intracellular glucose concentration, ATP concentration, lactic acid concentration, cell cycle, and genes associated with the cell cycle in MCF-7 cells.

We believe that combination of DOX with SGLT2i may reduce tumor cells’ resistance to DOX thereby make DOX more lethal to tumor cells.

This work aimed to examine the impact of the co-administration of CAN and DOX, DAP and DOX, and EMP and DOX on Breast tumor cells in an *in vitro* setting. Additionally, we assessed the cytotoxicity of these combinations in MCF-7 cells. Furthermore, we assessed the probable underlying mechanism impacting these factors such as intracellular glucose concentration, ATP concentration, lactic acid concentration, cell cycle, and genes associated with regulation of the cell cycle in MCF-7 cells.

## Materials and Methods

### Cell culture and treatment

MCF-7 human breast cancer cells were cultivated in Dulbecco’s Modified Eagle Media (DMEM) containing 10% fetal bovine serum (FBS) and 1% penicillin–streptomycin. The DMEM was bought from BIS BioTech, Jeddah, KSA, while the FBS was purchased from Sigma in St. Louis, MO, USA. The penicillin–streptomycin solution was from MOLEQULE-ON® in Auckland, New Zealand. All examined cells were kept in humidified incubator conditions of 37°C and 5% CO_2_ [16].

### Cytotoxicity assay

The MCF-7 cells were cultured until they reached a confluence of 60%–70% prior to treatment. The cytotoxicity of the test compounds was evaluated over a period of 48 h, with final concentrations between 0.16 and 80.0 µM. Similarly, DOX was evaluated at final concentrations between 0.01 and 5.6 µM. Additionally, combinations of the test compounds were assessed at a final concentration of 40 µM, along with DOX at final concentrations ranging from 0.01 to 5.6 μM. Subsequently, resazurin was introduced with a final concentration of 50 µM, followed by an incubation period of 3 h under controlled conditions of 37°C and 5% CO_2_ (CO_2_ incubator, model CCL-170B-8; ESCO life sciences, Singapore) in a humidified environment. The quantification of rezorufin was conducted by measuring its fluorescence at an excitation wavelength of 555 nm and an emission wavelength of 585 nm (SpectraMax Paradigm Multi-Mode Microplate Reader, Molecular Devices, CA, USA), with a cut-off at 570 nm for the detection of signals above background noise. The fluorescence measurement was performed using a bottom-read approach [17].

### Determination of the intracellular doxorubicin accumulation in MCF-7 cells

To quantify the intracellular concentration of doxorubicin, the MCF-7 cells were subjected to a treatment duration of 3 h using CANA (500 μM) (Cayman chemical, USA; Cat# 11575), EMPA (500 μM) (Cayman chemical, USA; Cat#17375), and a combination of DAPA (500 μM) (Cayman chemical, USA; Cat# 11574) with doxorubicin (100 μM) (Sigma-Aldrich, USA; Cat# D1515). After the incubation period, the cells were centrifuged (at a force of 200 g for 5 min) and subsequently subjected to two washes using 1 ml of phosphate-buffered saline (PBS). Following centrifugation, 0.2 ml of PBS was introduced. Then, the contents of the tubes were placed into 384-well plates at a volume of 20 μl per well. The concentration of the cells in each well was 10 × 103 cells. Control wells contained PBS without any cells as well as cells that were not treated. These control wells were used to measure the background fluorescence signal. Then, a volume of lysis buffer (0.1% Triton X-100 final solution) equal to the volume present in each well was added (20 μl of lysis buffer added to the 20 μl contents of the wells). After inducing cell lysis for two minutes on an orbital shaker, the plate was left to incubate for ten minutes at room temperature (25°C) in order to stabilize the fluorescence signal [18].

The data were expressed as averages ± SDs (n = 4). The fluorescence intensity of the untreated cells was calculated by subtracting the background fluorescence signal blank (only PBS w/o cells). The fluorescence intensity of the samples with treated cells was calculated by subtracting the signal of the untreated cells as a background.

### Determination of the intracellular ATP production in MCF-7 cells

First, 10 μM ATP was prepared in a culture medium. Then, serial 2-fold dilutions of ATP in the culture medium were prepared, and Cell Titer-Glo® Reagent equal to the volume present in each well (50 μl of Reagent added to wells containing 50 μl) was added. To stabilize the luminous signal, the samples were mixed for two minutes on an orbital shaker and then incubated for ten minutes at room temperature (25°C). The data were expressed as averages ± SDs in duplicate. The luminescent intensity of the samples with ATP was calculated by subtracting the background luminescent signal blank (only medium w/o ATP).

A CellTiter-Glo® Luminescent Cell Viability Assay was used to quantify the amount of ATP present, a sign that there were metabolically active cells present, to ascertain the number of viable cells in a culture.

After adaptation and adherence, MCF-7 cells were treated for 3, 6, or 24 h with either CAN (40 μM), EMP (40 μM), and DAP (40 μM) or CAN (40 μM), EMP (40 μM), and DAP (40 μM) combined with DOX (0.35 μM).

Following the incubation, the plates with cells were spun down (at 1000 g for 10 min). After centrifugation and the collection of the supernatant, a volume of CellTiter-Glo® Reagent equal to the volume present in each well (50 μl of Reagent added to wells containing 50 μl) was added. After combining the contents for two minutes on an orbital shaker to cause cell lysis, the plate was left to incubate for ten minutes at room temperature (25°C) in order to stabilize the luminous signal.

### Determination of glucose concentrations

Human breast adenocarcinoma MCF-7 (ATCC, HTB-22) cell line was provided by Promega, USA. MCF-7 cells were treated for 3, 6, or 24 h with either CAN (40 μM), EMP (40 μM), and DAP (40 μM) or CAN (40 μM), EMP (40 μM), and DAP (40 μM) combined with DOX (0.35 μM). The extracellular glucose levels were measured using a commercial Glucose (Glu) Colorimetric assay kit (God-Pod Method) (Elabscience, USA; Cat# E-BC-K234-M). The enzyme working solution (300 μl) was added to all required wells. Then, the 30 mmol/L standard solution (3 μl) or a sample (3 μl) was added to the wells and incubated at 37°C for 15 min. An analysis of the data was performed using a Spectra ax Paradigm Multi-Mode Microplate Reader (Molecular Devices, CA, USA) at a wavelength of 505 nm.

### Determination of Lactic acid concentrations

After adaptation and adherence, MCF-7 cells were treated for 3, 6, or 24 h with either Canagliflozin (10–40 μM), Empagliflozin (10–40 μM), Dapagliflozin (10–40 μM), and Doxorubicin (0.09–0.35 μM) or Canagliflozin (40 μM), Empagliflozin (40 μM), and Dapagliflozin (40 μM) combined with Doxorubicin (0.35 μM). Following the incubation, the plates with cells were spun down (at 1000 g for 10 min). After centrifugation, the supernatant (25 µl from each well) was collected and stored at −80°C for the determination of the extracellular levels of lactate.

### Cell cycle assay

Following a 24-h treatment period, the cells that had been treated were subjected to a cell cycle evaluation. In summary, the cells that were treated and the control cells were collected and washed two times with PBS. The cells were then treated with 70% ethanol that had been chilled on ice and then kept in an incubator with a temperature setting of −20°C for thirty minutes. The fixed cells were centrifuged at a speed of 5000 rpm for 5 min for ethanol removal. Following this, the cells were washed with PBS and ultimately resuspended in PBS. The cells were subjected to staining using 5 microliters of propidium iodide (PI) and thereafter were incubated for a duration of 30 min in dark conditions. The target cells were subjected to a flow cytometry analysis for the determination of the distribution of the cells across the different phases of the cell cycle. The cell cycle assay was conducted with a sample size of 10,000 cells per event, in triplicate, using an Amnis flow cytometer [19].

### Real-time quantitative PCR

A Pure Link RNA Isolation Kit, manufactured by Haven Scientific, Kingdom of Saudi Arabia, was utilized to extract total RNA from cancer cells that had been subjected to treatment, as well as their respective control samples, following the instruction manual’s procedure. The production of complementary DNA (cDNA) was achieved using a high-capacity cDNA reverse transcription kit (Applied Biosystems, USA). The objective of this study was to investigate the alterations in gene expression between cells that had been treated and cells that had not received any treatment. Using cDNA in triplicate, RT qPCR was used to identify variations in the expression of different genes. Table 1 shows a list of the primers used in this study.

**Table 1:** Primers list for q-RT PCR

Gene	Forward primers	Reverse primers
BAK	TTACCGCCATCAGCAGGAACAG	GGAACTCTGAGTCATAGCGTCG
BOK	ACGCCTGGCTGAGGTGTGCG	AGGAACGCATCGGTCACCACAG
BAX	ACCACAACCACACTCTGGAGGA	TCGGTTTCTGGTCTGGATGCCT
BCL2	ACTGGGCTGGTGGAGTCTTT	AACATCGCTACCAGGCCGAT
P16	CTCGTGCTGATGCTACTGAGGA	GGTCGGCGCAGTTGGGCTCC
P63	CAGGAAGACAGAGTGTGCTGGT	AATTGGACGGCGGTTCATCCCT
GAPHD	GTCTCCTCTGACTTCAACAGCG	ACCACCCTGTTGCTGTAGCCAA
CDK2	CCATCAGCACAGTTCGTGAGGT	TCAGTTCGGGATGTGGCACAGA

### Statistical analysis

All information was triple-transcribed and shown as means ± SEMs. The statistical significance was determined using Student’s 2-tailed *t-*test. A *p*-value of less than 0.05 was deemed statistically noteworthy. To perform the statistical analysis, Graph Pad Prism 8.0 was used.

## Results

### Cytotoxicity

**Figure 1:**
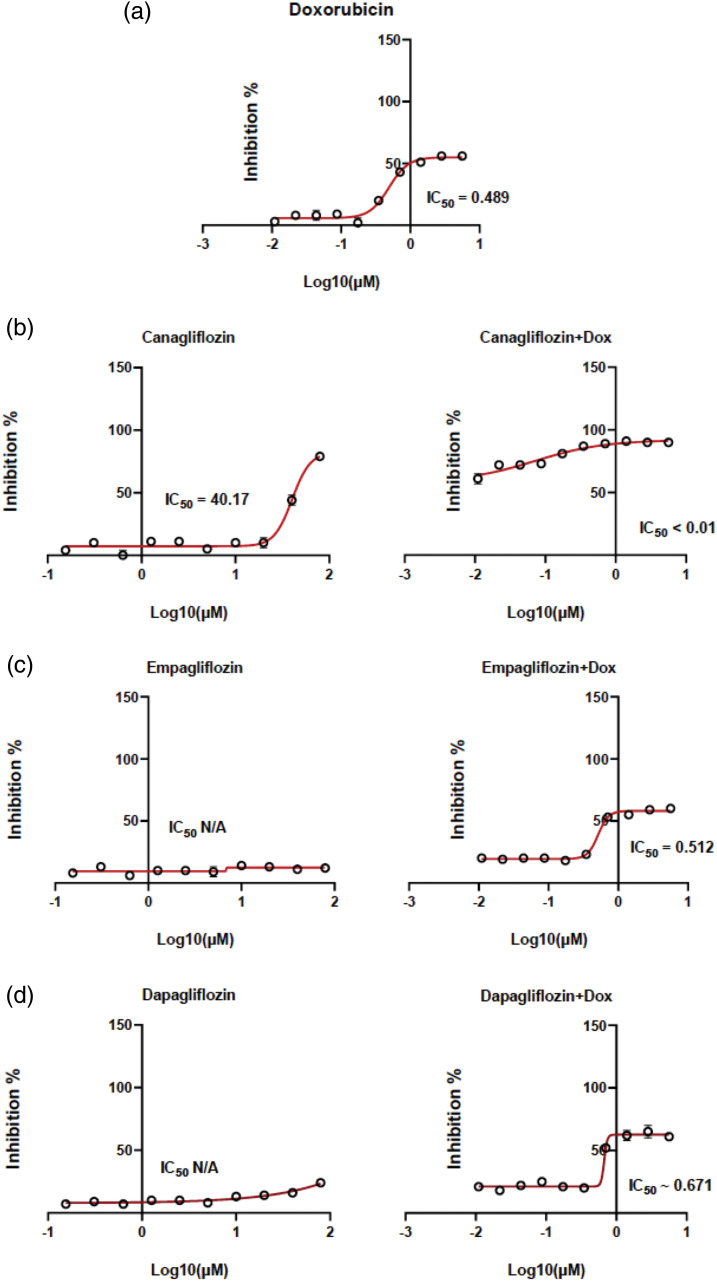
Dose–response curves. MCF-7 cells were exposed to (a) DOX, (b) CAN alone (left), CAN+DOX (right), (c) EMP alone (left), EMP+DOX (right), (d) DAP alone (left), and DAP+DOX (right) for 48 h, followed by the resazurin assay to determine cell viability (means ± SDs, n = 4).

The cytotoxicity data and the IC50 values of DOX CAN, EMP, and DAP were determined by constructing dose–response curves (Figs. 1a–1d). CAN exhibited moderate cytotoxicity (Fig. 1b) and increased the cytotoxicity of DOX in the MCF-7 cell line. The remaining test compounds (Figs. 1c, 1d) did not induce any significant cytotoxicity against the MCF-7 cell line and did not interfere with the cytotoxicity of DOX after treating the cells in combination with DOX.

### Doxorubicin accumulation

The aim of this study was to determine the effects of the compounds CAN, EMP, and DAP on the intracellular DOX accumulation in MCF-7 cells.

**Figure 2:**
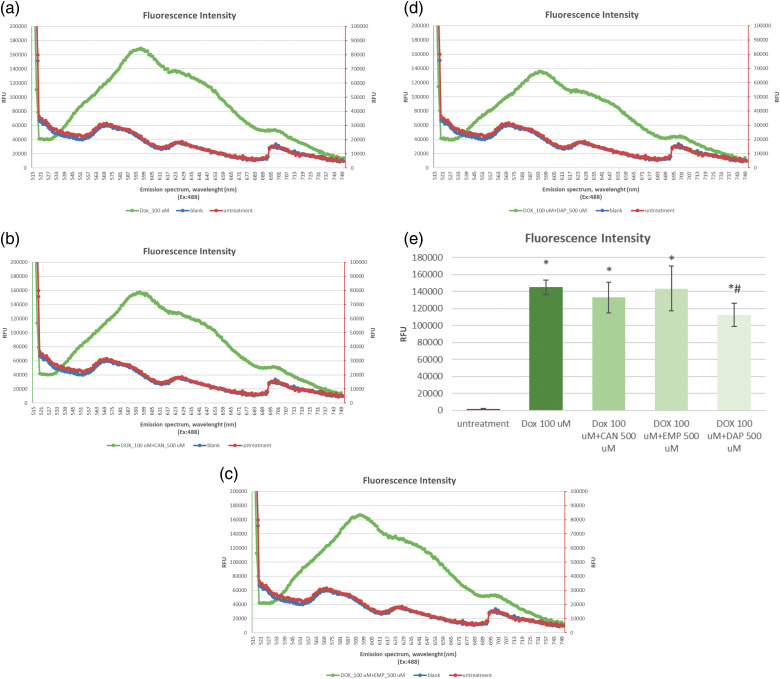
The emission spectra of intracellular fluorescence intensity of (a) DOX at a final concentration of 100 µM, (b) CAN+DOX at final concentrations of 100 and 500 µM, (c) EMP+DOX at final concentrations of 100 and 500 µM, and (d) DAP+DOX at final concentrations of 100 and 500 µM, respectively, in MCF-7 cells. (e) Intracellular fluorescence intensity of DOX alone and combined with other drugs. The data are expressed as means ± SDs (n = 4). *p < 0.001 *vs*. untreated cells, #*p* < 0.01 *vs*. doxorubicin alone.

The data on the accumulation of intracellular DOX alone (Fig. 2a) and DOX combined with CAN (Fig. 2b), EMP (Fig. 2c), and DAP (Fig. 2d) are presented in Fig. 2.

The compounds CAN and EMP did not induce increased accumulation of doxorubicin in MCF-7 cells. DAP (Fig. 2e), at the tested concentration, contributed to the opposite effect; it decreased the accumulation of intracellular DOX.

### Intracellular ATP production in MCF-7 cells

**Figure 3:**
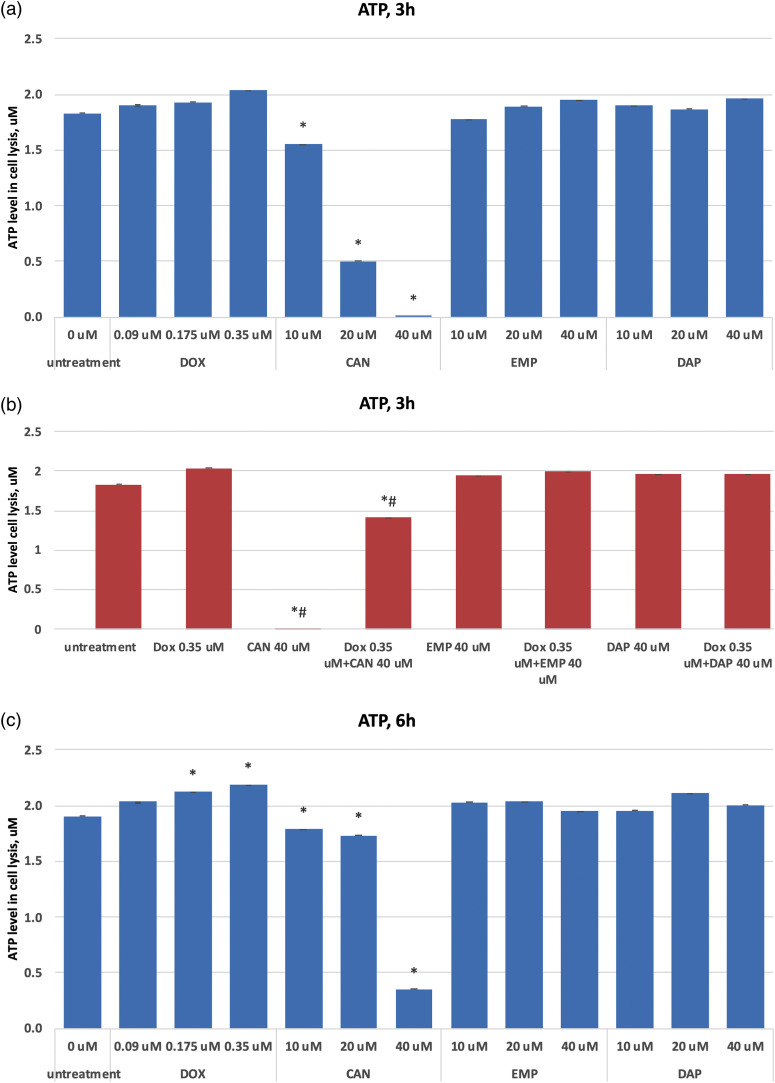

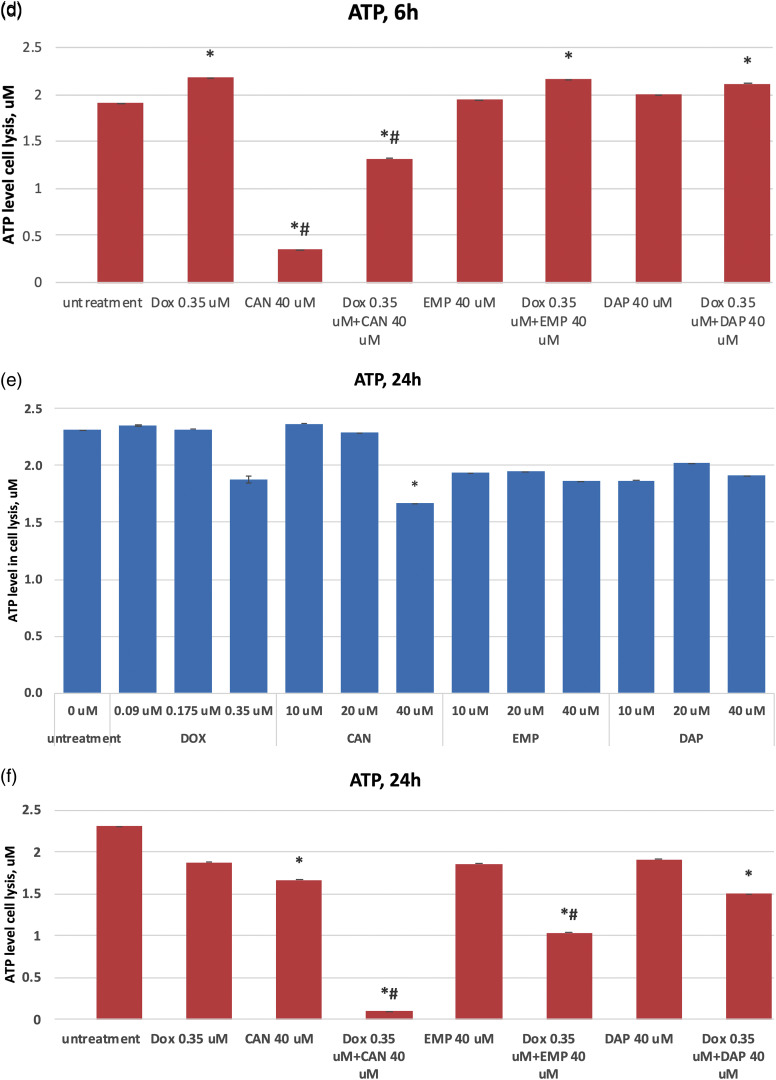
(a) Dose-dependent effects of DOX, CAN, EMP, and DAP on intracellular ATP production in MCF-7 cells after 3 h of incubation. (b) Effects of DOX, CAN, EMP, and DAP alone and CAN, EMP, and DAP combined with DOX on intracellular ATP production in MCF-7 cells after 3 h of incubation. (c) Dose-dependent effects of DOX, CAN, EMP, and DAP on intracellular ATP production in MCF-7 cells after 6 h of incubation. (d) Effects of DOX, CAN, EMP, and DAP alone and CAN, EMP, and DAP combined with DOX on intracellular ATP production in MCF-7 cells after 6 h of incubation. (e) Dose-dependent effects of DOX, CAN, EMP, and DAP on intracellular ATP production in MCF-7 cells after 24 h of incubation. (f) Effects of DOX, CAN, EMP, and DAP alone and CAN, EMP, and DAP combined with DOX on intracellular ATP production in MCF-7 cells after 6 h of incubation (mean ± SD, n = 4). *p < 0.05 *vs.* untreated cells (0 μM), #p < 0.05 *vs.* DOX-treated cells (0.35 μM)

Based on the quantification of ATP, which denotes the presence of metabolically active cells, the CellTiter-Glo® Luminescent Cell Viability Assay counts the number of viable cells in a culture. The number of cells in a culture directly relates to the amount of ATP. The ATP data after treating MCF-7 cells with CAN, EMP, DAP, and doxorubicin are presented in Figs. 3a–3f. The test compound CAN dose-dependently decreased the ATP levels in MCF-7 cells after 3 h (Fig. 3a), 6 h (Fig. 3c), and 24 h (Fig. 3e) of incubation. In contrast, DOX did not decrease the levels of ATP after 3 h (Fig. 3a) or 24 h (Fig. 3e) of incubation, and a dose-dependent increase in intracellular ATP production was observed after 6 h (Fig. 3c) of incubation. CAN (40 µM) combined with DOX (0.35 µM) significantly reduced the intracellular ATP levels *vs*. DOX alone (Figs. 3b, 3d, 3f). In addition, EMP (40 μM) combined with DOX (0.35 μM) decreased ATP levels *vs*. DOX alone after 24 h of incubation (Fig. 3f). DAP did not show similar results (Fig. 3f).

### Extracellular level of glucose in MCF-7 cells

**Figure 4:**
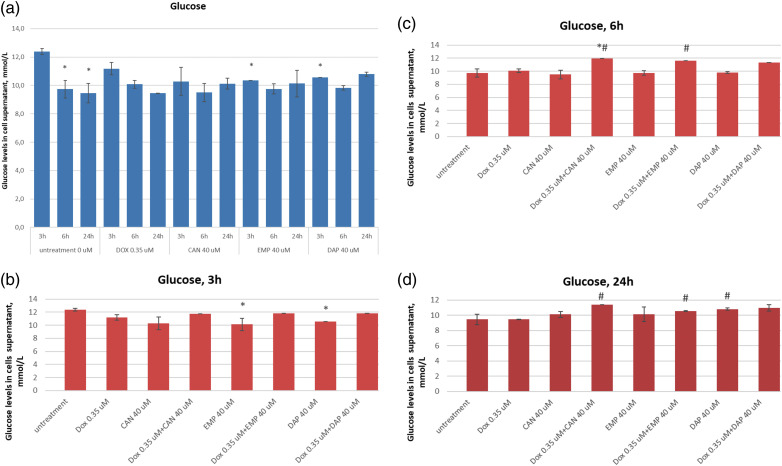
(a) Time-dependent effects of DOX, CAN, DAP, and EMP on extracellular level of glucose in MCF-7 cell supernatant. (b) Effects of DOX, CAN, DAP, and EMP alone and CAN, EMP, and DAP combined with DOX on extracellular level of glucose in MCF-7 cell supernatant after 3 h, (c) 6 h, and (d) 24 h of incubation (mean ± SD, n = 2). #*p* < 0.05 *vs*. DOX-treated cells (0.35 µM), *p < 0.05 *vs*. untreated cells (0 µM).

The purpose of this study was to test these compounds on a human cancer cell line and measure the extracellular glucose levels. The extracellular glucose data after treatment with canagliflozin, empagliflozin, dapagliflozin, and doxorubicin are presented in Fig. 4. The compounds did not inhibit glucose intake in a time-dependent manner at concentrations of 0.35 µM (DOX) or 40 µM (CAN, EMP, and DAP) (Fig. 4a). CAN (40 µM) and EMP (40 µM) combined with DOX (0.35 µM) did not inhibit glucose intake *vs*. Dox after 3 h (Fig. 4b), however it significantly inhibited glucose intake *vs*. DOX alone after 6 h (Fig. 4c) and 24 h (Fig. 4d) of incubation. DAP did not show similar results (Figs. 4c, 4d).

### Extracellular level of lactic acid in MCF-7 cells

**Figure 5:**
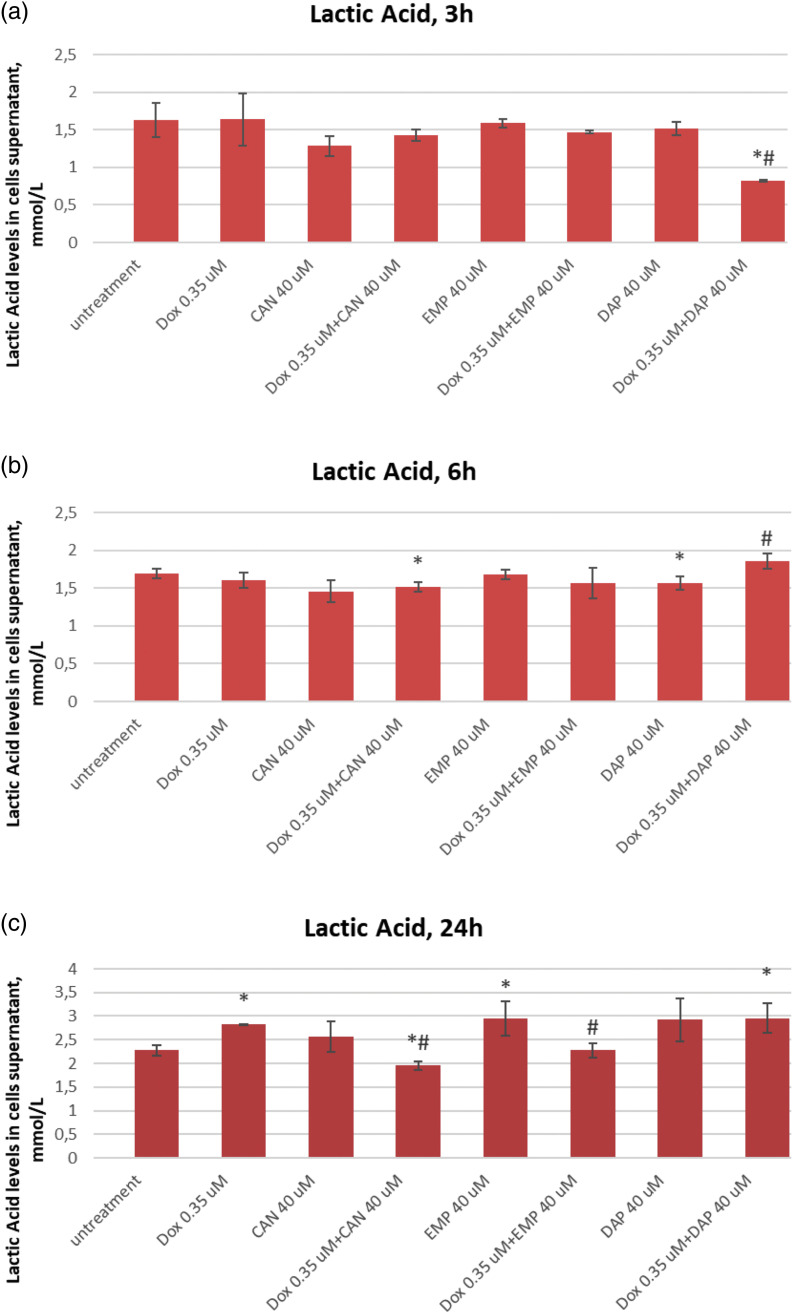
Effects of DOX, CAN, DAP, and EMP alone and CAN, DAP, and EMP combined with DOX on extracellular level of lactic acid in MCF-7 cells after (a) 3 h, (b) 6 h, and (c) 24 h of incubation (mean ± SD, n = 3). *p < 0.05 *vs*. untreated cells (0 µM), #*p* < 0.05 *vs*. DOX-treated cells (0.35 µM).

The extracellular lactic acid data after treatment with canagliflozin, empagliflozin, dapagliflozin, and doxorubicin are presented in Fig. 5.

DAP significantly decreased the level of lactate in DOX-treated cells after 3 h of incubation (Fig. 5a). The test compound CAN decrease lactate levels in a dose-dependent manner after 6 h of incubation (Fig. 5b) with MCF-7 cells. CAN (40 µM) combined with DOX (0.35 µM) and EMP (40 μM) combined with DOX (0.35 µM) significantly reduced the extracellular lactate level *vs*. DOX alone after 24 h of incubation (Fig. 5c).

**Figure 6:**
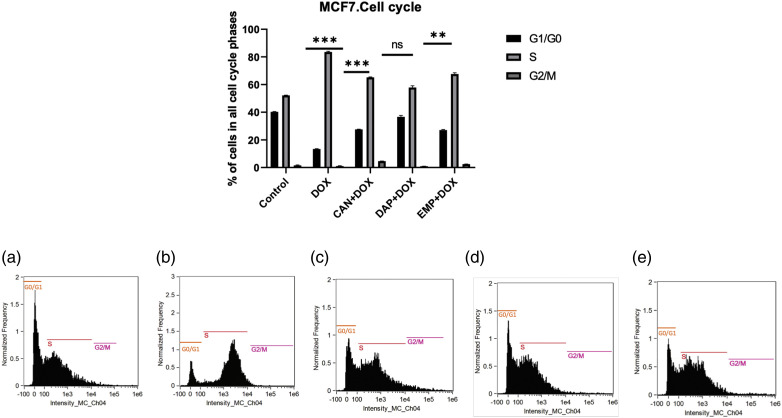
Cell cycle analysis of treated MCF-7 cells. (a) Control, (b) DOX, (c) CAN+DOX, (d) DAP+DOX, (e) EMP+DOX. Each bar represents the mean ± SEM (n = 3). ns: Not significant; **p < 0.01; ***p < 0.001.

SGLT2 inhibitors induce cell cycle arrest in MCF-7 cells. Since we noticed remarkable changes, we then asked whether these compounds induce cell cycle arrest. As we know, controlling the cell cycle is an important part of how breast cancer grows and spreads. Therefore, we conducted a cell cycle analysis. The combination of CAN+DOX arrested the cell cycle in the S phase with a small change in G1/G0 (Fig. 6). This was also the case for the EMP+DOX combination, but in case of DAP+DOX there were no changes in any phase of the cell cycle.

### RT-PCR results

**Figure 7:**
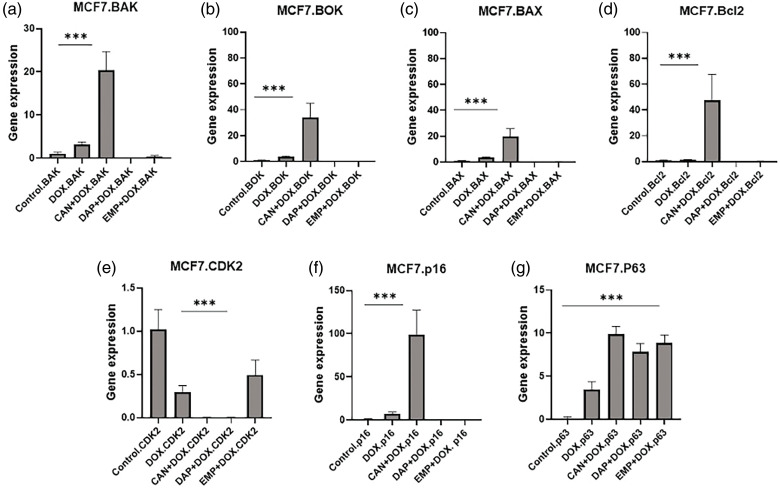
Gene expression analysis of MCF-7 cells treated with DOX alone, CAN+DOX, DAP+DOX, and EMP+DOX: (a) BAK, (b) BOK, (c) BAX, (d) Bcl2, (e) CDK2, (f) p16, (g) p63. Each bar represents mean ± SEM (n = 3). ***p < 0.001.

Since there were changes in the cell cycle, we performed RT-PCR to study the genes that are involved in the cell cycle (Fig. 7). We analyzed the expression of the BOK, BAK, BAX, and Bcl2 genes. Interestingly, the CAN+DOX combination significantly upregulated (Figs. 7a–7d) these genes, but no changes were observed for the other drugs.

There was a significant downregulation of CDK2 (Fig. 7e) and a small change in p16 in the case of DOX alone and a significant upregulation in the case of CAN+DOX (Fig. 7f), but no changes were observed for the other drug combinations. p63 was upregulated (Fig. 7g) in all cases.

## Discussion

Cancer, being a prominent contributor to global mortality rates, continues to be a subject of extensive scientific investigation in the medical field. This study primarily focuses on the discovery and evaluation of novel therapeutic interventions. Nevertheless, the process of developing new medications typically entails a protracted duration involving the identification of potential candidates and subsequent clinical validation. The repurposing of medicines that exhibit anticancer qualities represents a viable strategy for advancing cancer treatment. This is particularly true for drugs that have already been extensively utilized in clinical settings and possess well-documented safety profiles [20].

Recent research has indicated that CAN, DAP, and EMP have potential non-hypoglycemic benefits, such as cardiovascular protection [21], reduction of inflammation [22], and inhibition of tumor growth. Further, it was also reported that the combination of SGLT2i with DOX has a cardioprotective effect [23] In the current investigation, the co-administration of the medications CAN and DOX demonstrated heightened antitumor efficacy and a modest augmentation of the cytotoxicity of DOX. Additionally, it has been suggested that CAN may be effective in addressing drug resistance caused by DOX, a well-established chemotherapeutic agent, through the inhibition of P-gp activity. Cancer cells exhibit an increased demand for glucose in order to support their proliferation. SGLT2 is a transporter responsible for actively facilitating glucose uptake into cancer cells. This transporter has been identified in multiple tumor types. In a previous study, it was confirmed that MCF-7 cells showed significantly higher SGLT2 gene expression [24]. By suppressing SGLT2, it is possible to limit the proliferation of cancer cells. Additionally, on the viability of cancer cell lines, an MTT assay was carried out using an MCF-7 cell line. Various concentrations of SGLT2 inhibitors like canagliflozin, ipragliflozin, dapagliflozin, cisplatin, doxorubicin, and raloxifene were tested and their IC50 was determined [25] which is quite higher as compare to our study. In a previous study conducted by Nasiri et al., it was demonstrated that canagliflozin exhibits a dose-dependent effect, reducing glucose absorption in breast cancer cells [26]. Furthermore, the drug was found to effectively decrease tumor cell division *in vitro*. Canagliflozin, but not dapagliflozin or empagliflozin, exhibited an off-target, and thus SGLT2-independent adverse effect, characterized by the dual inhibition of glutamate dehydrogenase (GDH) and complex I of the mitochondrial electron transport chain (ETC) at pharmacologically relevant concentrations. This combined ETC and GDH inhibition obstructed glutamine input into the tricarboxylic acid (TCA) cycle (i.e., glutamine anaplerosis). As proliferating cells are much more dependent on anaplerosis, this dual inhibition explains why canagliflozin is significantly more toxic for proliferating than for quiescent cells and considerably more potent than classical ETC inhibitors [27]. In our study, it was observed that the combination of CAN and EMP, when administered with DOX, effectively suppresses glucose absorption. It is worth noting that the combination of CAN and EMP, when used in conjunction with DOX, resulted in a notable decrease in the ATP levels in MCF-7 cells. This finding aligns with a previous study by Kaji et al., which revealed a similar reduction in ATP levels but only in the context of the CAN and DOX combination [14]. Lactate plays a crucial role in cancer cells, as it can be readily taken up and metabolized by tumor cells to support the tricarboxylic acid (TCA) cycle. Additionally, lactate acts as a signaling molecule that regulates the cellular response to hypoxia. Previous research by Lee et al. demonstrated that lactic acid promotes the growth of tumor cells [28]. In this study, the combination of the compounds CAN, DAP, and EMP with DOX was shown to effectively inhibit cancer cell growth, thereby providing a means to control their proliferation.

One of the basic processes behind carcinogenesis is aberrant cell cycle progression, making regulators of the cell cycle machinery effective anticancer therapeutic targets. According to Liu et al., therapeutic interventions targeting the components of the cell cycle machinery have the potential to not only inhibit the proliferation of cancer cells but also to reverse the metabolism of cancer and reinstate cancer immune surveillance [29]. The concurrent administration of CAN+DOX and EMP+DOX effectively halts cell cycle progression during the S phase. Notably, only CAN+DOX demonstrates a significant increase in p16 gene expression, which is recognized as a tumor suppressor gene [30]. Activation of p16 leads to the inhibition of the cyclin-dependent kinases CDK4/6 and CDK2, which play a vital role in cell cycle regulation and are implicated in various physiological processes like cancer, tissue regeneration, aging, cellular senescence, and development [31]. Consequently, the increase in p16 or its baseline expression cannot be exclusively attributed to aging and senescence, as it may instead indicate the suppression of the cell cycle. Dysregulation of the cell cycle and abnormal activation of CDKs are present in nearly all human cancers [32] In a similar vein, it has been observed that all drugs exhibit a notable increase in the expression of the p63 gene, which is commonly believed to serve as a tumor suppressor protein. The expression of p63 is often increased in human breast carcinomas. This suggests that p63 plays a significant role in the development of tumours [33]. Jeon and colleagues demonstrated that when a human mammary epithelial cell line was exposed to doxorubicin and ubiquitin-like protein ISG15, it resulted in the ISGylation of the p63, hence increasing the sensitivity of doxorubicin to chemotherapy [34]. Given the crucial role of CDKs in various biological processes that are frequently dysregulated in cancer cells, pharmacologically targeting CDKs is seen as a promising approach for cancer treatment [35]. This upregulation is thought to occur through the transactivation of a group of genes, ultimately leading to the induction of cell cycle arrest and cell apoptosis [36]. According to Guo et al., findings from mouse models provide evidence indicating that p63 has the ability to suppress both carcinogenesis and metastasis [37].

Doxorubicin is a potent DNA-damaging drug that strongly stimulates the production of p53 and activates the transactivation of p21CIP1/WAF1 (p21). The p53 pathway is strongly implicated in the cellular response to DNA damage, as supported by multiple lines of evidence [38]. Furthermore, p53 mutations have been associated with the advancement of prostate cancer and the development of resistance to androgen regulation [39]. The cessation of cell proliferation is frequently initiated by a continuous DNA damage response and is traditionally carried out through the activation of the p16-Rb (retinoblastoma) and/or p53-p21 pathways. p16INK4a controls the G1 arrest that is dependent on the retinoblastoma protein [40].

The process of apoptosis has been found to promote the death of tumor cells, and it is highly regulated and controlled. Cells can commit suicide via a process called apoptosis, which can be initiated by signals from the outside (extrinsic apoptosis) or from within the cell itself (intrinsic apoptosis) [41]. Unregulated cell growth, as seen in malignancies, is linked to insufficient apoptosis. One of the most significant alterations in a cell that leads to cancer is that it does not die when it ought to. Proliferation and apoptosis, as well as antiapoptosis and proapoptosis processes, work together in normal breast cells to keep cellular homeostasis in check. Once the equilibrium is broken, unchecked cell proliferation, treatment resistance, and the recurrence of cancer cells might result from an overactive antiapoptotic signal pathway or a lack of signaling via the proapoptotic apoptosis route. The combination of CAN+DOX significantly upregulated the BAK, BOK, and BAX genes, which are proapoptotic [42]. The BAX and BAK proteins, which are crucial components of the B-cell lymphoma-2 (BCL-2) family, exhibit a response to several apoptotic triggers by permeating the outer mitochondrial membrane [43]. BCL-2 ovarian killer (BOK) exhibits a sequence similarity of approximately 70%–80% with BAX and BAK since it possesses BH1-3 domains and a carboxy-terminal transmembrane domain [44,45]. Upon transient upregulation, BOK induces cellular apoptosis, akin to the highly expressed proteins BAX and BAK [46]. The overexpression of BOK leads to the manifestation of classical apoptotic features, including the release of cytochrome c, the activation of caspase-3, the fragmentation of the nucleus and DNA, and the induction of cell death.

Study limitations the presence of SGLT2 in breast cancer and the expression of AMPK/mTOR are crucial factors for investigating the mechanism of action of the combination, which was not examined in the present study.

Altogether, in this study, a combination of DOX with SGLT2i drugs, specifically CAN+DOX, had a significant impact on the MCF-7 cell line and requires further exploration.

## Data Availability

The data and materials used in the present study are available from the corresponding authors upon reasonable request.
